# The Mental Health in Austrian Teenagers (MHAT) Study: design, methodology, description of study population

**DOI:** 10.1007/s40211-018-0273-2

**Published:** 2018-06-15

**Authors:** Michael Zeiler, Gudrun Wagner, Julia Philipp, Martina Nitsch, Stefanie Truttmann, Wolfgang Dür, Andreas Karwautz, Karin Waldherr

**Affiliations:** 1 Department for Child and Adolescent Psychiatry, Eating Disorders Unit, Medical University of Vienna, Waehringer Guertel 18–20, 1090 Vienna, Austria; 2 Ludwig Boltzmann Institute Health Promotion Research, Vienna, Austria; 3 FernFH Distance Learning University of Applied Sciences, Zulingergasse 4, 2700 Wiener Neustadt, Austria; 4 Department of Sociology, University of Vienna, Rooseveltplatz 2, 1090 Vienna, Austria

**Keywords:** Study population, Mental health disorders, Adolescents, Two-stage design, Epidemiology, Studienpopulation, Psychische Störungen, Jugendliche, Zweistufiges Design, Epidemiologie

## Abstract

Profound epidemiological data on the prevalence of mental health disorders and respective risk and protective factors is a prerequisite for adequate prevention, intervention and service planning. Children and adolescents are regarded as high priority groups for prevention in this field because of the high chronicity and individual burden of mental health disorders. The Mental Health in Austrian Teenagers (MHAT)-Study is the first epidemiological study based on a large representative sample of adolescents (*N* > 3700) in Austria in order to obtain the prevalence of a wide range of behavioral problems and psychiatric disorders. Adolescents are recruited from multiple settings (schools, course providers for early school leavers and psychiatric clinics) in order to enhance the representativity of the sample. A “gold-standard” two-stage design (screening questionnaire and diagnostic interviews) is used to obtain psychiatric diagnoses that are based on the 5th edition of the Diagnostic and Statistical Manual of Mental Disorders which was published by the American Psychiatric Association in 2013. This paper aims at presenting the study design and methodology of the MHAT study, describing the study population as well as discussing relevant strengths and limitations.

**Electronic supplementary material:**

The online version of this article (10.1007/s40211-018-0273-2) contains supplementary material, which is available to authorized users.

## Zusammenfassung

Fundierte epidemiologische Daten hinsichtlich der Prävalenz von psychischen Störungen und deren Risiko- und Schutzfaktoren sind eine Voraussetzung für die Planung von geeigneten Präventions- und Interventionsmaßnahmen sowie für die Versorgungsplanung. Kinder und Jugendliche haben aufgrund der hohen Chronizität von psychischen Erkrankungen und des Leidensdrucks der Betroffenen hinsichtlich der Prävention Priorität. Die Mental Health in Austrian Teenagers (MHAT) Studie ist die erste epidemiologische Studie, die anhand einer großen repräsentativen Stichprobe (*N* > 3700) von Jugendlichen in Österreich die Prävalenz von einer Bandbreite an Verhaltensproblemen und psychischen Störungen erfasst. Die Jugendlichen wurden aus verschiedenen Settings (Schulen, Kurse für frühe SchulabbrecherInnen, psychiatrische Einrichtungen) rekrutiert, um die Repräsentativität der Stichprobe zu erhöhen. Psychiatrische Diagnosen basierend auf den 2013 von der American Psychiatric Association veröffentlichten DSM-5 Kriterien wurden anhand eines zweistufigen Studiendesigns (Screening-Fragebogen, diagnostische Interviews) erhoben. Ziel dieses Artikels ist die Beschreibung des Studiendesigns und der Methodik der MHAT-Studie, die Darstellung der Studienpopulation sowie die Diskussion der relevanten Stärken und Limitationen.

## Introduction

Psychiatric disorders are regarded as one of the most burdensome diseases due to their high prevalence, early onset, high chronicity and low treatment rates causing significant individual, familial, economic and societal burden [1, 2]. Life-time prevalence estimates of any psychiatric disorder ranges from 12 to 50% in adult samples [1]. For children and adolescents, reviews and meta-analyses report point-prevalence estimates of any psychiatric disorder between 13 and 33% [3–5]. The onset of psychiatric disorders often occurs in adolescence [6] and mental health problems manifesting in childhood and adolescence often persist until adulthood [7–9] which underlines childhood and adolescence as priority life stages for research.

Several national and international policy papers have therefore emphasized the importance and necessity of mental health prevention and corresponding interventions starting early in childhood and adolescence [10–12]. Recently, a consortium of public organizations led by the Austrian Federal Ministry of Health who issued ten major health targets emphasized the importance of child- and adolescents’ health promotion in general and specifically the promotion of their psychosocial health [13].

A solid data base on the frequency of mental health disorders as well as on relevant risk and protective factors is a prerequisite for adequate prevention, (early) intervention and service planning. National prevalence figures are of great importance because of limited comparability of countries due to socio-cultural differences and existing, nationally different, mental health prevention programs.

By now, no epidemiological data on mental health and psychiatric disorders based on a representative sample of Austrian adolescents are available. Most studies on adolescents’ mental health carried out in Austria focus on specific subpopulations, like deaf adolescents [14–16], homeless adolescents [17] or children of chronically ill parents [18, 19]. Existing studies using school samples were either based on a small number of participants (e. g. [20]) and/or limited to a specific region [21, 22]. Thus, none of the prevalence figures presented in those studies can be generalized to the population of Austrian adolescents. Besides, the great majority of those studies used screening questionnaires only, e. g. the Strengths and Difficulties Questionnaire [23] or the Youth Self-Report [24]. In the biggest health study of adolescents in Austria, the Health Behavior in School-Aged Children (HBSC) Study [25], mental health is covered only by a few basic questions. Only a few studies used standardized clinical interviews in order to obtain diagnoses based on ICD or DSM diagnostic criteria [16, 17].

This short review of available data reveals a lack of knowledge regarding the prevalence of psychiatric disorders in childhood and adolescence in Austria.

However, epidemiological studies on psychiatric disorders in childhood and adolescence are not only of national interest. In 2013, the American Psychiatric Association published the 5th edition of the Diagnostic and Statistical Manual of Mental Disorders, DSM 5 [26], including significant changes in diagnostic criteria of mental disorders especially relevant for children and adolescents compared to the previous version (DSM-IV-TR). In particular, new diagnoses were included like Binge-Eating Disorder (BED) and Disruptive Mood Dysregulation Disorder (DMDD). Non-Suicidal Self-Injury (NSSI), Suicidal Behavioral Disorder (SBD) and Internet Gaming Disorder are for now being mentioned in the section of conditions for further study. Until now, there is no large epidemiological study on psychiatric disorders in childhood and adolescence that is based on DSM 5 diagnostic criteria including a wide range of mental health problems. This fact might be of special interest considering that the present study includes 27 psychiatric diagnoses within 9 diagnostic groups that are assessed by using the recently published DSM 5 criteria. Moreover, research on risk and protective factors for psychiatric disorders fosters the development of suitable prevention measures and contributes to early detection of mental health problems across national borders.

This paper aims to present objectives, design and methods of the Mental Health in Austrian Teenagers (MHAT) study, to describe the study population and obtained sample and to discuss strengths and limitations of the study design.

## Objectives of the MHAT study

The MHAT study was initiated to collect the first epidemiological data on adolescents’ mental health based on a large representative sample of 10–18 years old adolescents in Austria. The main objectives are as follows:

1. Obtaining the prevalence of general psychopathology and psychiatric disorders based on DSM-5 criteria in childhood and adolescence.
2. Identifying risk and protective factors for mental health problems and psychiatric disorders.

Furthermore, the MHAT study also aims to obtain data on mental health service use and contributes to several methodological considerations, for example the feasibility of online mental health assessments as well as the sensitivity and specificity of mental health screening instruments.

## Study design

A two-stage design is applied for the present study starting with a mental health screening in the first stage followed by diagnostic interviews in the second stage [27–29]. For the screening stage, inclusion criteria were defined as follows:

- students in the 5th, 7th, 9th and 11th school grade of all school types (except for special needs schools)
- children/adolescents aged 10–18 years in child- and adolescent psychiatry institutions for inpatient care and not attending a regular school
- adolescents aged 15+ years attending training courses for unemployed youth/early school leavers

Children and adolescents who do not have essential intellectual skills and essential German language skills were excluded from participation.

This multi-setting-recruitment strategy (school sample and non-school sample) was chosen in order to enhance the representativity and generalizability for the population of Austrian adolescents.

**Fig. 1:**
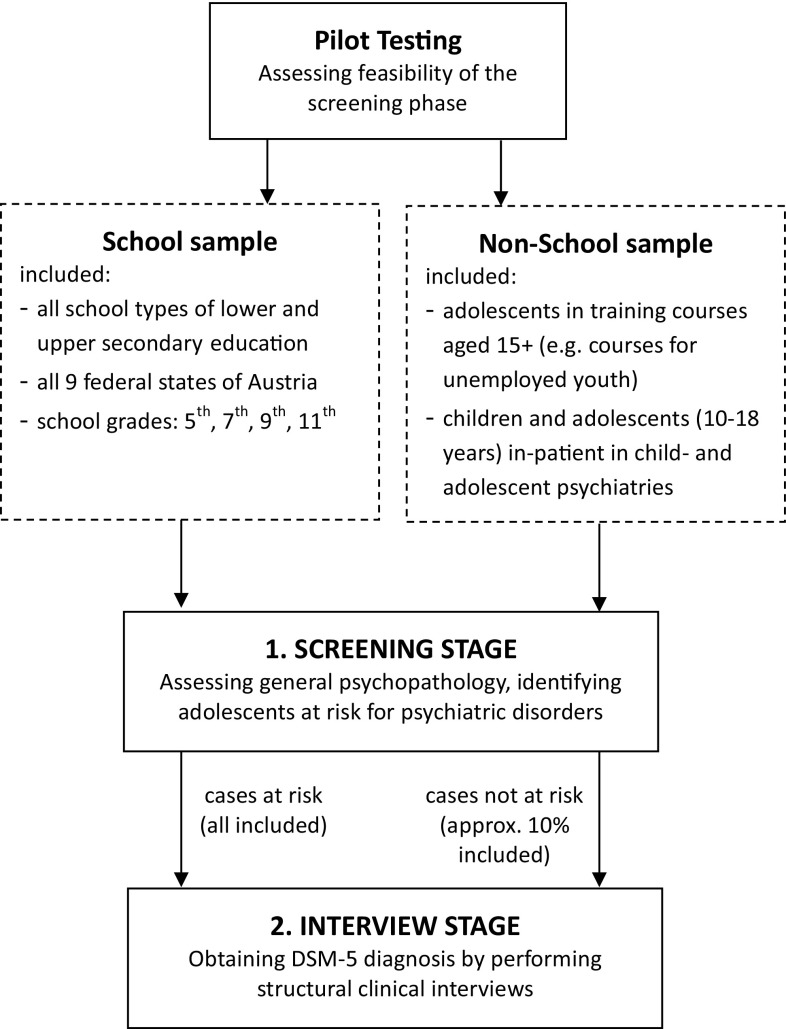
Study design of the Mental Health in Austrian Teenagers (MHAT)-Study

During the screening stage, questionnaires were given to the study participants to assess general psychopathology aiming at identifying adolescents at risk for psychiatric disorders. “At risk” is defined as scoring above the clinical cut off in at least one of the syndrome scales of the Youth Self-Report (YSR) [24] and/or scoring at least two points in the SCOFF [30] screening for eating disorders including at least intentional vomiting or significant weight loss. Details on the used screening instruments are described below. All adolescents who were screened “at risk” as well as a random sample of participants who scored below the predefined cut-off were invited to participate in the second stage during which a structural diagnostic interview was conducted (mainly by telephone) with the adolescents themselves and, whenever possible, a parent. The second stage aims at obtaining the prevalence estimates of psychiatric disorders based on DSM 5 diagnostic criteria. Main results of a pilot study, primarily aiming at evaluating feasibility and acceptability of the screening stage, were published in 2014 [31]. The study design is summarized in Fig. 1.

## Sample size calculations

A cluster sample of school classes is drawn. Sample size calculations for the cluster sampling were based on the following assumptions: 1. According to reviews on the prevalence of psychiatric disorders in childhood and adolescence which were available at the time of sample size calculations [4, 5, 32], the overall prevalence ranges between 10 and 20%. Thus, for the subsequent calculations, a prevalence of 20% is assumed, because it is the most conservative assumption concerning the required sample size. 2. According to Sullivan [33], a number of 30 clusters should be appropriate if the prevalence is between 10 and 90%, and a number of 60 clusters will improve the precision of estimates perfectly good. Therefore, a cluster size of 60 was intended per school grade. 3. The prevalence of any psychiatric disorder should be estimated with a precision of ±5%. 4. The finite population correction is ignored as a correction would have negligible effects. 5. The design effect (variance of *p* in a cluster design divided by the variance of *p* using a simple random selection) was set to 2 in accordance with common practice of conducting health surveys [34]. The required sample was finally calculated using Eq. 1 provided by Sullivan [33], with *n* representing the required sample size per school grade and sex, *deff* the design effect, *t* the t‑value for the used alpha level (0.05) and planned number of clusters (*m*), *p* the estimated prevalence and *q* the respective complementary probability and *d* the intended precision.

1 $$n=\textit{deff}\times \frac{t_{1-\frac{\alpha }{2},m-1}^{2}\hat{p}\hat{q}}{d^{2}}$$

The resulting sample size is 502 per school grade and sex. Including four school grades, the planned total sample size is 4016.

Since not all adolescents attend a regular school, the respective proportion of the planned total sample size of *N* = 4016 should be recruited from non-school institutions.

According to prior epidemiological studies, 17% of children and adolescents with a psychiatric disorder are in medical care and absent from school for a longer period of time [35] which corresponds to 3% (*n* = 16 per sex and age group) of the planned sample size. Those adolescents are recruited from mental health service institutions (child and adolescent psychiatries) in Austria.

In Austria, education is compulsory for nine years. According to the national report on education [36], 7.2% of adolescents who have already completed compulsory schooling, do not receive education any more. As there might be an overlap with those adolescents who are in inpatient care with mental health conditions, 4% of the sample of 11th graders (*n* = 20 per sex) are recruited from training courses for early school leavers and unemployed adolescents. The inclusion of those adolescents should contribute to the representativeness of the obtained results for the population of adolescents in Austria and counteract an underestimation of the prevalence by using a school sample only.

## Instruments

A complete list of instruments used and variables assessed in the screening and interview stage are available in Table 1. The main instruments are described in further detail below:

**Table 1:** List of included instruments and assessed variables

Questionnaires/Measurements	Contents
*Screening Stage*	
Youth Self-Report (YSR)	General Psychopathology, Behavioral and emotional problems
SCOFF	Screening Tool for Eating disorders
KIDSCREEN (KS) 52/27/10	Health-related Quality of Life (HrQoL)
	General HrQoL (KS-10), Self-Perception (KS-52), Parent Relation & Home Life (KS-52), Social Support & Peers (KS-27), School Environment (KS-27), Bullying (KS-52)
Headache Questionnaire	Migraine, Tension type Headache
Family Affluence Scale (FAS I, II)	Socioeconomic status
Sociodemographic variables	Sex, school grade, school type, Austrian state, class repetition, birth months and year, country of birth, parental country of birth, other people who live in the same household, occupation of parents, availability of an attachment figure
Other health-related variables	Current height & weight, self-perception of weight, diagnosed chronic somatic and psychiatric diseases (including onset of disease, use of medication, necessity of consulting health services regularly), diagnosed chronic somatic and psychiatric diseases of parents and siblings, life-time experiences of traumatic and burdening events
*Interview-stage*	
Kinder-DIPS	Structural diagnostic interview for psychiatric disorders in childhood and adolescents based on DSM-5 criteria including following disorders:
	*Disorders assessed from adolescents’ interview:* Specific Phobia, Social Anxiety Disorder, Generalized Anxiety Disorder, Panic Disorder, Agoraphobia, Obsessive-compulsive Disorder, Posttraumatic Stress Disorder, Major Depressive Disorder, Non-suicidal Self-Injury, Suicidal Behavior Disorder, Anorexia Nervosa, Bulimia Nervosa, Binge-Eating Disorder, Internet Gaming Disorder; Screening for alcohol, nicotine and drug abuse, Screening for non-organic psychosis
	*Disorders assessed from parents’ interview:* Attention-Deficit/Hyperactivity Disorder, Oppositional Defiant Disorder, Conduct Disorder, Tic Disorders (Tourette’s Disorder, Persistent Motor or Vocal Tic Disorder, Provisional Tic Disorder), Separation Anxiety Disorder, Selective Mutism, Disruptive Mood Dysregulation Disorder, Enuresis, Encopresis, Pica, Rumination Disorder, Avoidant/Restrictive Food Intake Disorder
	*Severity of diagnosis* based on clinical judgment (rated on a scale from 0 to 9)
	*General assessment of the functioning level* based on clinical judgment (rated on a scale from 0 to 100)
Treatment history & Help seeking	Use of any mental health service, type of therapy, type of care (inpatient/outpatient), duration and frequency of therapy, satisfaction with treatment, perceived benefit of the treatment, use of medications, help/treatment seeking (yes/no), reasons why no help/treatment is sought
Additional variables assessed in adolescents’ interview	School problems, school absenteeism, media consumption (time spent with watching television, gaming on computer/game console/mobile phone, using internet/social media, contacting friends by phone/short messages/emails), life-time history of pregnancy (for girls from age 14), cramped living conditions, frequency and burden of family conflicts
Additional variables assessed in parents’ interview	*Sociodemographic information:* Age of parents, sex and age of siblings, family structure (people in the same household, civil status), highest educational attainment of parents, occupation of parents and working hours, occupation of mother in the first year of child, approximate household income, first language of child and language that is mainly spoken at home, relocation with child associated with change of school, adolescents’ affiliation to a religious community and exercise of religious beliefs
	*Childrens’ early development:* complications in pregnancy and during birth, Alcohol and nicotine consumption of mother during pregnancy, abnormalities in early childhood development, problems with speaking
	*Life-time history of chronic diseases and severe accidents*
Interview-Evaluation	Short qualitative interview regarding the preceding interview based on three levels 1. Content (of questions) 2. Feelings 3. Situation (Interviews by telephone)

The German version of the Youth Self-Report (YSR) [24] was used to assess general psychopathology. Several previous epidemiological studies have used the YSR for screening purposes [28, 37, 38]. Behavioral and emotional problems are assessed by 103 problem items on a 3-point scale. Items are summed up to eight syndrome scales (Cronbach alphas 0.56–0.86) and three second-order scales (Cronbach alphas >0.86). Convergence between the YSR syndrome scales and psychiatric diagnoses assessed from structured diagnostic interviews have been demonstrated (e. g. [39–41]). Raw scores are transferred into T‑Scores according to existing German norms. According to the manual, a cut-off of T > 63 was used for the second-order scales and a cut-off of T > 70 for the syndrome scales to identify adolescents scoring in the clinical range. The competence scales usually included in the YSR are omitted for this study.

The SCOFF is a screening instrument for eating disorders [30] and was included because the YSR does not cover eating disorder psychopathology. The German version was first used by Hölling and Schlack [42]. Core-features of eating disorders including intentional vomiting, loss of control over food, significant weight loss, body dissatisfaction and food intrusive thoughts are assessed. Dichotomous item-ratings are summed up to a total score (range 0–5) whereas a score ≥2 reflects an eating disorder risk. A high sensitivity and specificity of 100 and 87.5% for anorexia and bulimia nervosa is claimed by the authors [30].

The KIDSCREEN questionnaire [43] assesses various aspects of health-related quality of life. The scales used within the MHAT study are described in Table 1. Items are rated on a 5-point Likert scale. Scale scores are transferred to T‑scores according to the available Austrian norms for children and adolescents aged 8–18 years. Internal consistencies range from 0.77 to 0.89.

The Family affluence scale (FAS) [44] is a self-report measure to assess the family wealth and serves as a proxy for the socioeconomic status (SES). It was developed in the context of the Health Behavior in School-Aged Children (HBSC) study [25] as an alternative to standard measures of SES, like family income, parents’ occupation and education due to known difficulties for children and adolescents to report these variables adequately. Good criterion validity has been demonstrated by contrasting the FAS score with general measures of national wealth [44]. The FAS consists of four items assessing the number of cars owned by the family, if the participant has his own bedroom, number of holidays spent with the family in the last year and the number of computers owned by the family.

Additionally, the class teachers were asked to rate all students of their class (participating and not participating) on the basis of a few items. The questionnaire was created by the MHAT research team in order to assess a possible non-response bias. The following items were assessed: Sex and school grade, class repetition (yes/no), school absenteeism, effort during lessons, ability to concentrate (compared to other students of the same age), socially integrated in the class (rather yes vs. rather no), disciplinary problems (rather yes vs. rather no), withdrawn in school (rather yes vs. rather no) and making contact to parents or school council due to behavioral problems in the past (yes vs. no vs. no but it would be advisable).

The Kinder-DIPS [45] is a structured clinical interview for assessing psychiatric disorders in children and adolescents. It consists of two parallel interview guides (one for adolescents and one for parents). The unpublished version used by the MHAT team has been provided by Schneider and colleagues [46] and is based on the published version from 2009. It has been adapted for DSM 5 diagnostic criteria and two new sections were added for assessing NSSI and SBD. Three additional interview sections were developed by the Austrian research team and additionally included: Avoidant/Restrictive Food Intake Disorder (ARFID), Rumination Disorder and Internet Gaming Disorder. Significant clinical impairment is covered by the DSM 5 diagnostic criteria. All psychiatric disorders captured in the interview are listed in Table 1. To reduce the participants’ burden, the interview sections were divided between the adolescent’s and the parent’s interview. Psychiatric disorders that are mainly assigned to the internalizing domain were only assessed in the adolescents’ interview whereas disorders that are mainly assigned to the externalizing domain and mainly occurring in infancy and early childhood are only assessed in the parents’ interview. This allocation was made due to the fact that internalizing problems generally can be assessed with a higher validity in adolescents themselves than by parental reports and that externalizing problems are more valid when assessed by parents than by adolescents [45].

## Recruitment and data collection

### School sample

All Austrian schools at the lower and upper secondary educational level were initially contacted per e‑mail, invited for participation and asked to provide the actual number of school classes. If the schools did not reply, they were reminded up to three times and additionally contacted via telephone. Out of 2547 schools, 428 (16.8%) agreed to participate. Of those available schools a stratified cluster sample with school classes as clusters and school type and federal state as strata was drawn. The latest available national school statistics were used to obtain the number of classes and students per school grades, school types and federal states reflecting the population of students in Austria. A maximum of two classes per school were selected. Class teachers autonomously organized and moderated the data collection of the screening stage, including obtaining written informed consent from the adolescents and their legal representatives prior to data collection and delivering questionnaires to the participating students during one school lesson of 50 min. Feasibility for the teachers was checked in a pilot study [31]. Two versions of the questionnaire were available, an online version and an equivalent paper-pencil version. The online version was developed using the software Limesurvey [47]. An online assessment was preferred if a sufficient number of computers for the whole class was available. In total, 78% of the data were obtained using the online version.

Positive screened adolescents as well as a parent were further selected for a structured clinical telephone interview. Furthermore, from negatively screened adolescents a random sample of about 10% (as well as a parent) was selected for a structured clinical telephone interview. All telephone interviews were conducted by clinical psychologists and clinical psychologists in training under supervision. An interviewer training was conducted prior to the start of the interview stage consisting of a theoretical introduction and a practical training. The interview duration was about one hour for the adolescents’ and about 45 min for the parents’ interview on average. A guided computerized assistance system based on Microsoft® Access was used for the interview conduction and data entry. Participants who could not be reached received a message on their mailbox. If the participants did not contact the study team themselves, they were contacted at least two more times on different dates at different times of the day. After at least three unsuccessful contact attempts, the cases were coded as “not reachable”. The interviewers followed guidelines defining the procedures in case of self-endangerment. Contact information on appropriate mental health services were given to all participants suffering from a psychiatric disorder detected in the interview. Group supervision with interviewers was held regularly and was moderated by two clinical psychologists experienced in child and adolescent psychiatry and experienced with the used interview instrument. Individual supervision and case reviews were provided whenever needed.

### Non-school sample

Institutions which provide courses for unemployed adolescents were randomly selected from a list and asked for participation. Four institutions agreed, two in an urban and two in a rural region of Austria. In total, eight course classes were included. The procedure of data collection was equivalent to the school sample except for a member of the study team moderating the screening stage.

Eight out of twelve child- and adolescent psychiatry institutions in Austria in five different federal states were approached and agreed to participate. In each institution, a census of all children and adolescents aged 10–18 years in inpatient care was intended. The procedure of data collection was adapted due to the institutions’ needs. In any case, the study was first introduced to the clinic staff and the concrete procedure of data collection was discussed. In the next step, the study was introduced to the patients by the research group and both, the patients and parents were asked for informed consent. The screening stage was either organized and moderated by the clinic staff itself or by a member of the research group. Dependent on the patients’ needs and possibilities, the interview was either conducted in-person at the clinic or by telephone. The interviews with the parent were conducted by telephone in all cases.

## Ethics and data confidentiality

The study protocol was approved by the Ethical commission of the Medical University of Vienna and by the Federal Ministry of Education and Women’s Affairs. Data collection was in line with the Austrian data protection law. Major ethical aspects include: 1. Obtaining written informed consent from the adolescents and their legal representatives prior to the data collection of the screening stage, 2. providing a “hotline” to be used in case of any problems or psychological distress, 3. providing contact information for mental health services at the end of the telephone interview to anyone with detected current mental health problems and/or seeking help. Data confidentiality was ensured by the following procedures: 1. No personalized data were stored in an electronic database. Contact information (such as name and telephone number) were administered offline only and only by medical and psychological staff. All interviewers were clinical psychologists or clinical psychologists in training under supervision and had to sign a confidentiality agreement. The interviewers were blind with regard to the screening results of participants. 2. Screening and interview data were matched by a code that does not allow drawing any conclusions about the individual and the participating institution. 3. SSL-encryption was used for the transmission of data obtained by the online questionnaire.

## Sample description and representativeness

**Fig. 2:**
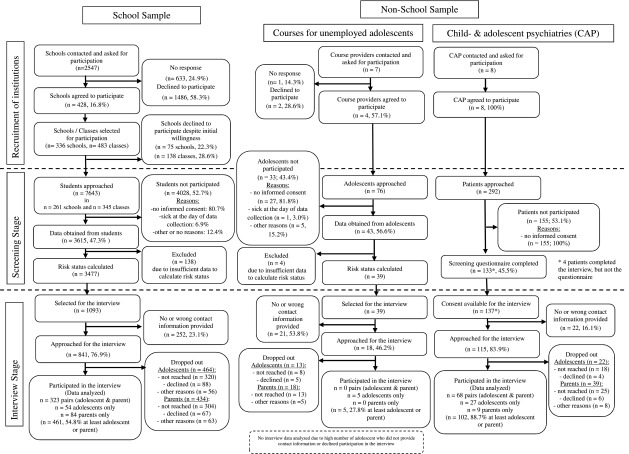
Flow chart depicting recruitment of the school and non-school sample

A detailed flow chart depicting the whole process of recruitment in the school and non-school sample is presented in Fig. 2. There was a low general participation rate of schools (16.8%), a medium participation rate of course providers for unemployed adolescents (57.1%) and a 100% participation rate of child- and adolescent psychiatry units. Regarding the individuals in the screening stage, there was a participation rate of around 50% for the school and non-school sample. Most individuals who did not participate provided no informed consent.

A detailed table on the distribution of participating students according to school grade, school type and federal state as well as the percentage of the planned sample size which was actually reached can be seen in Online Resource 1. In total, screening data from 3615 students were obtained which corresponds to about 94% of the sampling plan. Students of the 5th grade were underrepresented (58% of the planned sample size) because of the proportionally high refusal rates in this group, whereas 9th graders were slightly overrepresented (117% of the planned sample size). General and academy secondary schools were slightly underrepresented (77 and 85% of the planned sample size), polytechnical and vocational schools were slightly overrepresented (125 and 124% of the planned sample size). Regarding the Austrian federal states some were slightly over- or underrepresented, however, the sample size reached for Eastern‑, Middle- and West-Austria fit the sampling plan very well. Deviations from the sampling plan will be addressed by data weighting procedures. A description of the school and non-school sample including the most important demographic variables is depicted in Table 2. The proportion of participating students living in urban areas defined as a city with more than 10,000 inhabitants (58.2%) was slightly higher than the corresponding proportion in the general population living in urban areas (42.7%). However, data retrieved from students living in the federal capital or in the capital of a federal state (30.6%) perfectly fits the respective proportion in the general population (29.6%). The proportion of students with migration background (16.7%) was slightly lower than the proportion in the general population of adolescents under 15 years (24.4%). However, this gap is partially compensated by the inclusion of adolescents not attending a regular school where the proportion of adolescents with migration background is higher. Regarding the socioeconomic status, the proportion of participating students classified as “high” (72.8%) was higher than the respective proportion in the HBSC study [48] (60.9%). The self-evaluated financial situation of the family assessed with a 5-point scale was the same between the MHAT and the HBSC study (MHAT mean: 2.25; HBSC mean: 2.23).

**Table 2:** Sociodemographic characteristics of the study population

	School Sample	Non-School Sample	
	(*n* = 3615)	Courses for adolescents (*n* = 43)	Child- & adolescents psychiatries (*n* = 133)
*Females (%)*	55.3%	48.8%	78.1%
*Age (Mean, SD)*	14.53 (2.31)	18.23 (1.91)	15.28 (1.97)
*Place of residence* ^*a*^			
Urban area	58.2%	83.7%	100%
Rural area	41.8%	16.3%	0%
*Place of residence*			
Federal capital or capital of federal state	30.6%	83.7%	100%
Other place	69.4%	16.3%	0%
*Migration background*			
General^b^ (%)	25.4%	74.4%	32.3%
1st generation^c^ (%)	6.1%	65.8%	5.4%
2nd generation^d^ (%)	10.6%	7.9%	6.2%
*FAS I—Score* ^*e*^ * (Mean, SD)*	6.45 (1.73)	3.55 (2.10)	5.67 (1.82)
*FAS II—Score* ^*f*^ * (Mean, SD)*	8.93 (2.06)	5.44 (2.46)	7.86 (2.25)
*FAS category* ^*g*^			
Low (%)	2.0%	40.0%	3.8%
Moderate (%)	25.2%	42.5%	42.0%
High (%)	72.8%	17.5%	54.2%
*Self-evaluation of familial financial situation* ^*h*^ * (Mean, SD)*	2.25 (0.87)	2.81 (1.15)	2.62 (0.94)
*Parental employment status (% employed)*			
No parent	2.8%	42.5%	9.8%
One parent	19.1%	32.5%	24.1%
Both parents	78.1%	25.0%	56.1%
*Family status*			
Both biological parents	74.5%	32.0%	45.0%
Single parent	16.6%	40.0%	38.3%
Patchwork	9.9%	28.0%	16.7%
*BMI standard deviation score (Mean, SD)*	−0.05 (1.08)	−0.09 (1.16)	−0.52 (1.69)
*Diagnosed chronic somatic illness* ^*i*^ * (%)*	11.3%	18.4%	30.5%
*Diagnosed mental illness* ^*i*^ * (%)*	2.9%	16.2%	85.6%
*Diagnosed physical illness in family (parents/siblings)* ^*i*^ * (%)*	15.3%	8.8%	36.7%
*Diagnosed mental illness in family (parents/siblings)* ^*i*^ * (%)*	4.3%	22.9%	28.1%
*Any burdensome event in life (like death, accident)* ^*i*^ * (%)*	40.1%	47.1%	53.8%
*Any potential traumatic event in life (like abuse, violence)* ^*i*^ * (%)*	6.7%	45.7%	37.4%

^a^Urban area: Place with >10,000 inhabitants; Rural Area: Place with <10,000 inhabitants

^b^Own birth place or birth place of one parent in foreign country

^c^Own birth place in foreign country

^d^Own birth place in Austria, birth place of both parents in foreign country

^e^Family affluence scale (4-item version)—higher values indicate higher socioeconomic status

^f^Family affluence scale (6-item version)—higher values indicate higher socioeconomic status

^g^Family affluence scale—categorization (based on 4‑item version)

^h^Familial financial situation is rated on a 5-point rating scale (1 = very good, 5 = very bad)

^i^ Based on adolescents’ self-report

Regarding the interview stage, a significant amount of participants who were selected for the interview did not provide any contact information or provided insufficient or wrong contact information (23.1% of the school sample, 53.8% of the sample of courses for unemployed adolescents and 16.1% of the clinical sample). Within the school sample, in 54.8% of cases, at least the adolescent or a parent participated in the telephone interview whereas this participation rate was 88.7% in the clinical sample. Due to the high number of unemployed adolescents who did not provide contact information or declined participation, we have to refrain from analyzing interview data from this subsample.

## Discussion of strength and limitations of the MHAT study

MHAT is the first epidemiological study on adolescents’ mental health and psychiatric disorders based on a large population sample in Austria that uses a “gold standard” two-stage design. This design will provide a profound data basis on mental health problems of adolescents in Austria and will therefore add significant value to the field of psychiatric epidemiology in Austrian adolescents [49]. MHAT is the first large epidemiological study in Europe applying DSM 5 diagnostic criteria for obtaining prevalence estimates of a wide range of psychiatric disorders and it also includes disorders newly included in the DSM 5. The MHAT study pursues a multi-setting recruitment strategy by including adolescents who cannot be reached via the regular school system. Especially in the field of mental health it is important to approach also hard-to-reach groups of the population in order to increase representativeness of the sample. Another measure to counteract selection bias is the inclusion of a teacher’s questionnaire assessing basic data on mental health of both participating and non-participating students that goes beyond a simple assessment of demographic variables which can help to evaluate a possible non-response bias. This is an advantage towards studies using registers of residents were no health-related informations on non-participants are available. Furthermore, to counteract false negative screening results also a random sample of negative screened adolescents was selected for telephone interviews.

MHAT is one of the first large epidemiological studies on mental health that used online screening assessments for the majority of participants. Considering the rising significance and use of online assessments [50, 51] this study can also contribute to this field of research. First results regarding the eating disorder risk indicate that online assessment is comparable to paper-pencil assessment [52].

Some limitations of the MHAT study have to be mentioned as well: As the study has a cross-sectional design, all associations between mental health variables and other variables such as quality of life and potential risk and protective factors can only be interpreted in terms of correlations and not causally. Response rate of schools was very low. Many schools did not respond to our e‑mails and phone calls or mentioned time reasons and general work overload as reasons for non-participation. Another limitation pertains to the teachers conducting the screening phase at schools and not researcher of the project team. Although the teachers got a detailed instruction on how to moderate the data collection and feasibility of the procedure was confirmed [31], it might lack standardization across all schools. There was few or no personal contact between the project team and participating schools. If teachers were reminded more often to collect informed consent forms it might have influenced the relatively low response rate of about 50%. Diagnostic interviews were held over telephone. This procedure was chosen due to economic reasons. Even though interviewers were trained and a standardized interview guide was used, the lack of non-verbal information could have influenced the validity of obtained diagnoses. On the other hand, the use of telephone for diagnostic assessments might also have advantages over face-to-face assessments. The social distance between interviewer and interviewee might also foster more honest responses [53] and therefore help to increase the validity of obtained diagnoses. The interview sections of the Kinder-DIPS were divided between the adolescent’s and the parent’s interview. The adolescents received questions primarily dedicated to the internalizing spectrum whereas parents received questions primarily about the externalizing spectrum. Regarding the validity of psychiatric diagnoses, the best procedure would have been to give all interview sections to both, the adolescent and the parent, and to merge their answers afterwards in order to obtain the diagnoses. However, this was not possible since the duration of an interview should not exceed one hour.

## Conclusion

The two-stage design, the large sample size of more than 3700 adolescents and the use of DSM 5 diagnostic criteria itself would provide a sound basis for a study on the epidemiology of psychiatric disorders in Austria. The MHAT study provides a much more profound data basis by additionally applying a multi-setting recruiting strategy, including a teacher’s questionnaire and interviewing also the parents in the second stage. Thus, the MHAT study contributes greatly to the research on the burden of mental disorders among adolescents in Europe.

## Caption Electronic Supplementary Material

Online Resource 1: Actual number of students participated in the MHAT screening stage per school grade, school type and federal state and proportion (%) of the sample size reached compared to the sampling plan
