# Supplementary material for: The Mental Health in Austrian Teenagers (MHAT) Study: design, methodology, description of study population
Source: Neuropsychiatr. 2018 Jun 15;32(3):121–32. doi: 10.1007/s40211-018-0273-2 (PMC6132433; doi:10.1007/s40211-018-0273-2)
Supplement: Supplementary file 1 — Online Resource 1: Actual number of students participated in the MHAT screening stage per school grade, school type and federal state and proportion (%) of the sample size reached compared to the sampling plan [file 40211_2018_273_MOESM1_ESM.pdf]

Online Resource 1

Supplement to:
The Mental Health in Austrian Teenagers Study (MHAT): design, methodology, description of study population

Neuopsychiatrie

Michael Zeiler, Gudrun Wagner, Julia Philipp, Martina Nitsch, Stefanie Truttmann, Wolfgang Dür, Andreas Karwautz, Karin Waldherr

Corresponding author:
Karin Waldherr
Ferdinand Porsche Distance Learning University of Applied Sciences
E-Mail: karin.waldherr@fernfh.ac.at.

Online Resource 1: Actual number of students participated in the MHAT screening stage per school grade, school type and federal state and proportion (%) of the sample size reached compared to the sampling plan

		Austrian federal state											
School grade	School type	Vienna	Burgenland	Lower Austria	Upper Austria	Carinthia	Salzburg	Styria	Tyrol	Vorarlberg	Sum	Total	% of sampling plan
5th	General secondary school	41	30	91	70	24	10	82	32	7	387	563 (58%)	57,50
	Academic secondary school	38	19	44	29	7	0	34	0	5	176		58,86
7th	General secondary school	48	12	154	142	24	34	176	39	31	660	923 (95%)	96,92
	Academic secondary school	43	0	85	33	5	9	56	0	32	263		90,38
9th	Academic secondary school	21	0	90	62	16	15	13	0	33	250	1134 (117%)	89,93
	Polytechnical school	38	6	88	48	16	17	22	31	20	286		124,89
	Vocational school	88	2	164	115	26	75	61	49	18	598		128,60
11th	Academic secondary school	33	9	36	29	12	0	33	16	41	209	990 (107%)	110,00
	Vocational school	70	13	91	111	9	12	58	33	4	401		117,60
	Part-time vocational school[1]	78	0	105	66	12	0	71	9	39	380		95,72
	Sum	498	91	948	705	151	172	606	209	230	Total: N = 3610		
	% of sampling plan	70,04	71,09	133,71	101,59	58,53	61,21	113,91	61,47	120,42	93,91		

School types	Sum	% of sampling plan
General secondary school	1047	77,33
Academic secondary school	898	84,88
Polytechnical school	286	124,89
Vocational school	999	123,95
Part-time vocational school[1]	380	95,72
Total	3610	

[1] with apprenticeship training

Note: As for 5 participants data on school grade and/or school type and/or federal state was missing, the total sample size is reduced from 3615 to 3610 in this table.
